# Experimental Assessment of the Transmission Performance of Step Index Polymer Optical Fibers Using a Green Laser Diode

**DOI:** 10.3390/polym13193397

**Published:** 2021-10-02

**Authors:** María Ángeles Losada, María Mazo, Alicia López, Candela Muzás, Javier Mateo

**Affiliations:** 1 Electronics Engineering and Communications Department, EINA, University of Zaragoza, María de Luna, 1, E-50018 Zaragoza, Spain; 704871@unizar.es (M.M.); aliclope@unizar.es (A.L.); 759549@unizar.es (C.M.); jmateo@unizar.es (J.M.); 2 Photonics Technology Group (GTF), Aragón Institute of Engineering Research, University of Zaragoza, Mariano Esquillor Gómez s/n, E-50018 Zaragoza, Spain

**Keywords:** polymer optical fibers, step-index, SI-POF, green light, 520 nm, optical communications

## Abstract

Large-core polymer optical fiber (POF) links have limitations in capacity and reach due to the fibers’ high modal dispersion and attenuation. Most of these links use red laser diodes, even though the attenuation spectrum of poly(methyl methacrylate) (PMMA), the basic polymer used to manufacture these fibers, has a lower minimum in the green region. Therefore, we set out to explore the potential use of green light in transmission systems, comparing the performances of three step-index polymer optical fibers (SI-POFs) with different numerical apertures. We obtained measurements of intensity distribution, frequency response and bit error rate (BER), as functions of fiber length. We have also compared the fibers’ frequency responses with red and green light for a few selected lengths. Our results confirm that SI-POFs attenuate less in response to green light, which can increase their length. This advantage is partially counterbalanced by a slightly higher dispersion that limits the capacity of the high-aperture fibers, particularly at relatively short lengths. Our conclusions are critical to understanding SI-POF behavior and to designing thorough SI-POF models that can aid the design of POF-based links for different scenarios.

## 1. Introduction

Polymer optical fibers (POFs) are fibers whose cores and claddings are made of plastic materials, of which poly(methyl methacrylate) (PMMA) and amorphous fluoropolymers are the most used [1,2]. These fibers have found a number of applications over the years, in which their use is advantageous compared to other media, including sensing and communications in short-range scenarios [3,4]. In particular, the use of POFs as a transmission medium in communication systems is strongly favored by their transmission properties, which are notably different for the fibers made of the two aforementioned materials. POFs made of amorphous fluoropolymers, also called perfluorinated POFs, have core diameters from 50 to 250 μm, and their transmission performance can even surpass that of multimode glass optical fibers [5]. One of the most common materials for this type of fiber is polyperfluoro-butenylvinylether (commercially known as CYTOP^®^), which gives rise to high performance fibers that, however, partially lose some of the advantages of standard large-core PMMA POFs. These fibers have larger core diameters (typically 980 μm) and numerical apertures (NAs) of up to 0.6 μm. They can be manufactured with a single core (SC) or a number of smaller cores (multi-core, MC) with different index profiles: step-index (SI), multi-step-index (MSI) or graded-index (GI) profiles [6]. In general, large-core PMMA POFs, which we refer to as standard POFs, have narrower bandwidth and higher attenuation than glass fibers, but are still adequate to meet the demands of short-range networks in automotive, industrial, domestic and more recently, avionics environments [7]. The automotive industry led the way, introducing them as the transmission media in car data links [8], and more recently, they have also succeeded in domestic networks, usually combined with Wi-Fi [9]. Nowadays, the manufacturing of PMMA fibers with thermoset polymer jackets able to sustain temperatures up to 105 °C, has raised the interest of the aeronautics industry in POFs [10].

In addition to the advantages of all optical fibers (electromagnetic immunity and electric passiveness), POFs’ specific characteristics make them very attractive. They are lightweight, flexible and resistant to impacts. Moreover, PMMA fibers experience minimum attenuation in the visible range of the spectrum (530, 570 and 650 nm), which facilitates failure detection and avoids safety issues [2]. Their most attractive features, however, are their large cores and high numerical apertures that enable easy connections using simple termination tools. In consequence, standard POFs can be easily manipulated and do not require expert handling. This “do-it-yourself” feature is responsible for POFs’ success in domestic applications [11], and their reduced deployment and maintenance costs constitute a key factor for cost-effective applications [12,13,14]. On the other hand, standard POFs’ large sizes and high apertures allow transmission of millions of modes, giving rise to high modal dispersion that decreases bandwidth, and thus limits POF systems’ capacities. In addition, PMMA has high diffusion coefficients that promote strong mode coupling with a negative impact on transmission properties [15].

Although the absolute minimum of PMMA’s spectral attenuation is near 530 nm, POF links are often based in laser diodes emitting in the red region of the visible spectrum because of the wider choice of commercially available devices. The use of sources at other wavelengths for data transmission was reviewed in [6]. Those transmitters were mainly based on green emitting LEDs that profited from the lower attenuation of POFs in this region to increase the reach of relatively low-capacity links. More recently, the application of spectrally efficient modulation formats has boosted system capacity, which can be further increased with wavelength division multiplexing (WDM) schemes that transmit in green light, among other wavelengths [16,17]. However, manufacturers only include data for the red region of the spectrum in their fiber characteristics sheets, and measurements of basic transmission properties at other wavelengths are scarce.

In this context, we aimed to explore the use of optical sources emitting in the wavelength range for which PMMA has minimum attenuation using a commercially available laser diode. In particular, we characterized three large-core PMMA fibers with different numerical apertures using a laser diode emitting at 520 nm. Measurements of received power, intensity distribution, frequency response and bit error rate (BER) for different fiber lengths provide a quantitative assessment of the potential of a fiber when working near the green light attenuation minimum, which is crucial in order to accurately estimate their applicability in different environments. Moreover, these measurements are indispensable to understanding POF behavior in order to postulate realistic models.

The paper is organized as follows. The methodology is addressed in the first section, where the experimental protocols and the set-up that integrated several independent subsystems to obtain the different measurements are outlined. In the next section, the experimental results are shown for all fiber types and fiber lengths. Transmission parameters extracted from the measurements, received power, spatial distribution width, frequency bandwidth and bit rate for a fixed BER, are represented against fiber length to compare the performances of the different fibers using green light. In addition, frequency responses for green and red light obtained under the same experimental conditions are compared. Finally, the conclusions obtained from the analysis of our results are summarized.

## 2. Materials and Methods

In this section, we describe first the experimental set-up composed by the individual subsystems to obtain the intensity angular distribution at the fiber output, the frequency response, the power budget and the overall transmission performance through the measurement of far field pattern (FFP) images, S_21_ transmission parameter, received optical power and BER, respectively. To obtain these measurements as functions of length under the same launching conditions, we used a protocol based on the cut-back method.

Secondly, we describe a control experiment devised to compare the behavior of the fibers with two wavelengths, 520 and 650 nm, under similar conditions.

### 2.1. Fibers and Experimental Set-Up

The three analyzed fibers, manufactured by Mitsubishi, were all step-index with 1 mm core diameter, but had different numerical apertures. MH4001 (MH), also called ESKA-MEGA, is a low-aperture fiber with a NA of 0.3. This lower aperture is achieved by a double-cladding design [6]. GH4002 (GH) is a duplex fiber with a NA of 0.5, also termed ESKA-PREMIER. Finally, BH4001 (BH), with the highest NA, 0.58, is a fiber resistant to high temperatures with a thermoset polymer jacket adequate for automotive or avionics applications.

**Figure 1:**
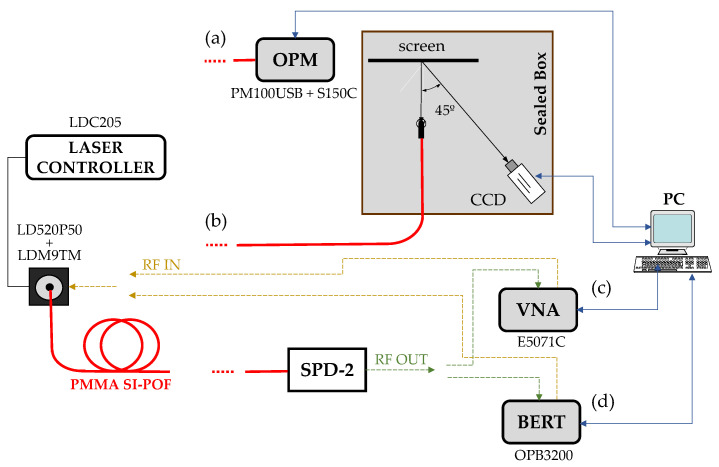
Experimental set-up with different subsystems to obtain (**a**) received optical power, (**b**) FFP images, (**c**) S_21_ transmission parameter and (**d**) bit error rate.

A diagram of the experimental set-up is schematized in Figure 1, where separate blocks labeled (a) to (d) were used to obtain the different measurements. As the components have been thoroughly explained elsewhere, we only give a very short description here [18,19].

The fiber input end was connected to the laser diode using a ST connector, and the output end was connected to a different device depending on the measurement being performed. The optical source used was the laser diode LD520P50 (LD520 in the following) with a nominal output power of 50 mW at 520 nm. The bias current was set to 80 mA at 25 °C, using the LDC205 laser controller and the LDM9T laser mount from Thorlabs. This laser mount was used to build an optical transmitter by directly modulating the laser diode either using the radio-frequency (RF) output of a vector network analyzer (VNA E5071C from Agilent), or using the data output of a BER Tester (OptoBERT^TM^ OPB3200 from Optellent. Inc., San Jose, CA, USA). Both for the frequency domain characterization and for the BER performance measurements, the receiver used was the SPD-2_650 (SPD) from Graviton with a nominal 3-dB bandwidth of 1.2 GHz, but with a good response of up to 3 GHz. Its responsivity is 1 mV/mW at 650 nm, and 0.6 mV/mW at 520 nm. The SPD RF output was connected either to the VNA to obtain the S_21_ transmission parameter or to the BER Tester to measure error-rates, as depicted in Figure 1c,d. The SPD also has an ST connector for the fiber. The received optical power was directly measured using an optical power-meter PM100USB with a S150C sensor, both from Thorlabs, connected to the fiber through a SMA connector, Figure 1a. Finally, as shown in Figure 1b, the output end of the fiber was placed in a VL connector at a distance of 7.5 cm from a white screen, to register the reflected FFP image by means of a 12-bit monochrome camera (QICAM FAST 1394CCD).

### 2.2. Experimental Methodology

The experimental protocol was the following: We started with the longest fiber length whose input end was cleaved, stripped and inserted into a ST connector. The connector end was polished using two pieces of sandpaper with different roughness levels, and connected to the laser mount, where it remained invariable throughout the duration of the experiment to ensure that all fiber lengths were tested under the same launching conditions. Then, we applied the same process to the output fiber end and connected it to the different devices to measure S_21_ parameter, optical power, FFP and BER. For each fiber length, the output end was cleaved, stripped and polished twice. The optical power and S_21_ parameter were measured at least five times, always changing the receiver or power-meter connection. Fifteen FFP images were registered to obtain an average. The BER was measured as a function of data rate for a basic OOK transmission using a pseudorandom binary sequence (PRBS) pattern length of 2^23^-1. When the whole set of measurements was completed, a fiber segment was cut from its output end to get a shorter fiber. The output end of the remaining fiber was prepared, and a complete set of measurements was repeated for that fiber length.

This protocol was applied to the three fibers considered, but starting from different fiber lengths depending on their different characteristics. This starting length was 130 m for MH, 150 m for GH, and 100 m for BH. All the analyzed fibers were reeled over a 20-cm spool. For the GH fiber, we used the advantage of the GH duplex reel to run this experimental protocol simultaneously for red and green light. One of the two fibers of the duplex was connected to the LD520 and the other to a red laser diode, LD650P007 (LD650, 7 mW at 650 nm), that was placed in another LDM9T mount driven by a LDC200 controller set to 35 mA at 25 °C. In this duplex experiment, the input ends of both fibers were fixed, and the output ends were connected sequentially to the different devices to obtain the complete set of measurements for both wavelengths at a given fiber length. The input ends of the two fibers of the duplex were split over a segment of 10 cm to permit the connection of each end in a different laser mount. At the output end, the split segment of the duplex fibers was only 3 cm, because the fibers were alternatively connected to the various devices.

We also designed a control experiment to determine if the fibers have different frequency responses depending on the wavelength. In this experiment, we fixed the launching conditions using a 15 cm ST-connectorized fiber pigtail to inter-connect one of the two LD sources to the tested fiber. The purpose of this launching technique was to provide an overfilled launch where the source optical power was coupled up to the fiber’s high-order modes or angles [6]. Conversely, direct injection, as achieved in the previously described cut-back method, is usually underfilled; i.e., optical power is concentrated only at low angles, and in addition, is prone to large variability [20]. The wider angular distribution provided by the pigtail is very stable and more independent of the source’s spatial characteristics. Thus, we measured the S_21_ transmission parameter with one end of the pigtail connected to one of the two LD sources, and with the other connected to one of the fibers. The other end of the tested fiber was connected to the receiver and remained connected when the pigtail input end was changed from the LD520 to the LD650. Thus, the detection conditions were exactly the same for the two wavelengths, and the variability of the input conditions was minimized by the indirect launch. This protocol was applied to the three fiber types using individual segments of three different lengths, 15, 20 and 50 m, and was repeated several times using the SPD receiver. In addition, another commercial receiver, SPA-2_650 from Graviton (SPA), was considered for the sake of completeness, which is intended for detecting light with higher numerical apertures than the SPD, guaranteeing that most of the optical power at the output of the fiber was captured. The SPA has the same responsivity as the SPD at the two wavelengths and a slightly lower frequency response with 3 dB bandwidth of 1 GHz that drops more abruptly than for the SPD.

## 3. Results and Discussion

This section is organized into two different subsections to display and discuss the results obtained applying the experimental protocols described above. First, we compare the transmission properties of the three analyzed fibers as functions of the fiber length using the green laser diode as the transmitter source. Then, the frequency responses obtained with the red and the green laser diodes under overfilled launching conditions are examined to assess the differences in transmission between both wavelengths.

### 3.1. Transmission Properties Using Green Light as a Function of Fiber Length

In this subsection we present a thorough characterization of the transmission properties of the fibers, including spatial properties, time/frequency properties and overall transmission performance. The spatial properties of light at the outputs of the fibers were characterized in terms of encircled angular flux (EAF) obtained from FFP measurements, and the fiber frequency response was calculated from measurements of the S_21_ transmission parameter of the link. Finally, the measurement of BER as a function of data rate completed the characterization, giving a full picture not only of the fiber but of the whole link. In order to ease the comparison of the different fibers, numerical parameters were extracted from these characterization measurements: optical power; EAF width; fiber 3-dB bandwidth; and maximum data rates for a given BER value were obtained as a function of fiber length and allowed us to evaluate the influence of the numerical aperture.

**Figure 2:**
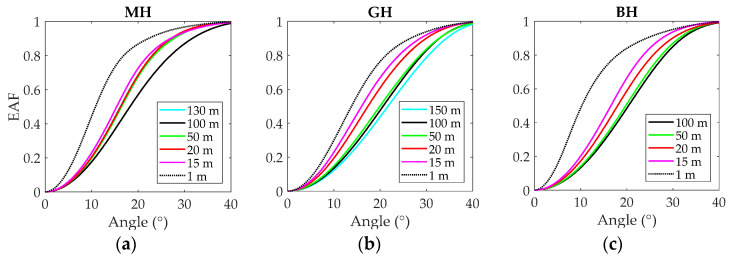
EAFs at selected fiber lengths. (**a**) MH fiber, (**b**) GH fiber and (**c**) BH fiber.

The EAF, obtained from the measured FFP, is a particularly interesting way to represent the spatial distribution of optical power because it represents the portion of power contained in a given solid angle [21]. Figure 2 displays the EAFs obtained for the MH on the left, for the GH in the middle and for the BH on the right, at several selected lengths: 100, 50, 20 15 and 1 m, and at the maximum measured length, which was different for each fiber.

The graphs illustrate the different behavior of the fibers. First, we can observe that the EAFs for the 1-m MH and the 1-m BH are very narrow, suggesting an underfilled launch, and the EAF for the 1-m GH is wider. As we said before, the direct launch is rather variable, and it is possible to get very different launching conditions depending on the fiber termination and connector position [20]. The EAFs for the two POFs with wider NAs, GH and BH exhibited steady widening with increasing fiber length, as should be expected by power diffusion [18]. For the longest fiber lengths, BH and GH EAFs only show small changes, which indicates that the equilibrium mode distribution was reached at the maximum measured length for both fibers. However, MH displayed different behavior: there is not a monotonous increase of EAF width with length, and the widest EAFs was found at 100 m rather than at 130 m. During the experiments, we observed that, particularly for this fiber, the end polishing produced relevant changes both in the output power and in the shape of the FFP, which introduced high variability over these measurements. We suggest that this behavior can be explained by alterations in its double-cladding structure incurred when applying the sandpaper.

**Figure 3:**
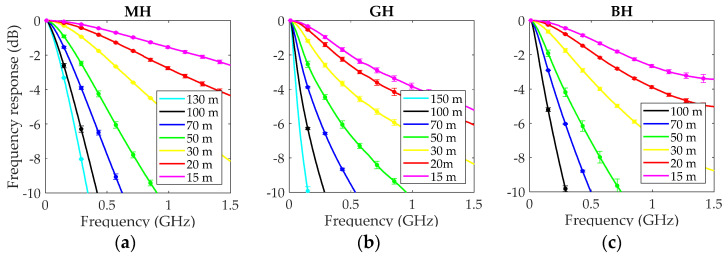
Normalized frequency response at selected fiber lengths. (**a**) MH fiber, (**b**) GH fiber, and (**c**) BH fiber.

As for the frequency response, it was obtained from the measurement of the S_21_ transmission parameter, for a given length L, according to:

(1) $$H_{L}\left(f\right)={\left(\frac{S_{21,\;L}\left(f\right)}{S_{21,\;\mathrm{REF}}\left(f\right)}\right)}^{\frac{1}{2}}$$

where $S_{21,\;L}\left(f\right)$ is the link transmission parameter measured for a fiber length L, and $S_{21,\;\mathrm{REF}}\left(f\right)$ is the transmission parameter for a 1-m fiber segment. This last measurement was taken at the end of the cut-back experiment under the same launching conditions, and was used as a reference to account for the frequency responses of the active devices. The frequency responses for the MH, GH and BH fibers are represented on the left, middle and right plots of Figure 3, respectively. The lines show the averages of five measurements, and the error bars their standard deviations. The graphs show selected lengths of all fibers used: 100, 70, 50, 30, 20 and 15 m. The frequency responses for the longest measured lengths are also shown for MH (130 m) and GH (150 m).

All graphs show that the frequency response narrowed with increasing fiber length by the effect of modal dispersion. A comparison of the graphs reveals that the MH had the flattest frequency responses at all lengths, which is consistent with its reduced NA of 0.3. The GH and the BH fibers displayed similar frequency responses, although the BH fiber responses were clearly better for 15 and 20 m. We argue that the cause of the difference is that the GH responses were measured with overfilled launch, which was made plain by its wider EAF for 1 m (Figure 2). In the overfilled launch, power is injected at high propagation angles, increasing modal dispersion, which degrades the frequency response, particularly for shorter fiber lengths.

**Figure 4:**
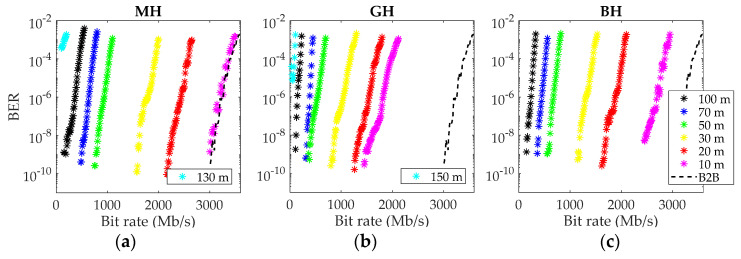
BER as function of bit rate in Mb/s at selected fiber lengths. (**a**) MH fiber, (**b**) GH fiber, and (**c**) BH fiber.

Finally, the measurements of transmission performance were carried out by analyzing the bit error rate for a simple data transmission, varying the data rate. Results in terms of BER versus data rate in Mb/s are shown in Figure 4 in the same way as in the previous figures. The curves are shown for the same lengths as for the frequency responses, except that the BER for 10 m is shown instead of that for 15 m.

For the three fibers, the link worked even for the longest measured length, although in the cases of MH and GH, only at very low bit rates and with a high BER value. When decreasing the fiber length, the curves were pushed to the right, and the link can work at higher bit rates. For a 10-m MH fiber, the link shows no penalty to the back-to-back (B2B) condition. The B2B curve was obtained with a very short fiber segment and describes the system’s limitation due to the transmitter and receiver. For the GH and BH fibers, link length has to be shortened to below 5 m to cancel the penalty, consistently with their lower frequency responses.

**Figure 5:**
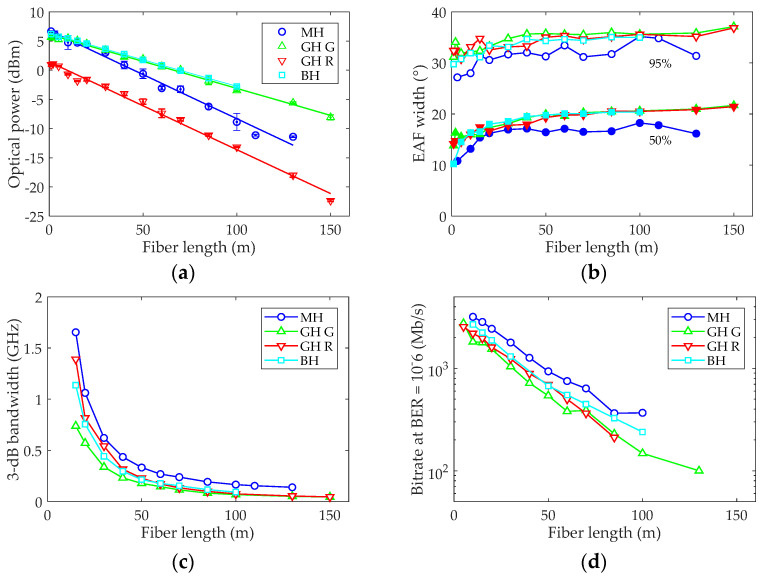
Transmission parameters as a function of fiber length for the MH, GH and BH fibers: (**a**) optical power; (**b**) 3-dB bandwidth; (**c**) EAF width at 50% and 95% power; (**d**) data rate for BER = 10^−6^.

Figure 5 illustrates the evolutions with length of the fiber transmission parameters extracted from the measurements obtained for all fiber types. In all graphs, the data obtained using the LD520 are represented for the MH fiber with blue circles; for GH with green upright triangles; and for the BH with cyan squares. To compare the properties obtained with red and green light, the data obtained for the other GH fibers in the duplex reel using the LD650 are represented with red inverted triangles.

In Figure 5a, received power versus fiber length is shown for the three fibers with the LD520, and for the GH additionally with the LD650. The symbols represent the averages and the error bars the standard deviations of the measurements. The straight lines show the linear fits to the experimental data. Their slopes represent the fiber attenuation at that wavelength and are: 0.09 dB/m for GH and BH, and 0.15 dB/m for MH at 520 nm. The attenuation obtained at 650 nm for GH is 0.15 dB/m, clearly above the value obtained at 520 nm. The attenuations at 650 nm for MH and BH, stated in their characteristics sheets, are 0.16 dB/m and less than 0.2 dB/m, respectively, which confirms the lower attenuation for all fibers at 520 nm. For MH, the difference is less significant than for the higher NA fibers, but data for this fiber have higher variability, as demonstrated by its larger error bars.

Figure 5b shows the EAF width for both 50% and 95% encircled power, as a function of fiber length. The graph shows how MH’s spatial distribution is the narrowest with an EAF width at 50% power of 16°, consistent with its lower aperture, whereas for the other fibers, this value is slightly above 20°. The differences between GH and BH, and between the red and green results for GH, are not significant. Notice that the width increase was quite smooth for the measurements obtained for GH and BH, but had an irregular trend for MH that we attribute to the effect of end polishing over its double-cladding structure.

Figure 5c,d shows the 3-dB bandwidth and the data rate for a BER of 10^−6^ as functions of fiber length, respectively. Both graphs illustrate how the MH fiber presents superior performance with the highest bandwidths and bit rates for all fiber lengths, consistently with its reduced modal dispersion due to its lower NA. In fact, this fiber can support Gigabit data rates at up to 50 m. However, error-rates below 10^-6^ cannot be obtained with this fiber above 100 m, because of the low received power due to its higher attenuation. For all fibers, the maximum data rates with BER of 10^−6^ are higher than 100 Mb/s at 100 m, and the maximum is exactly this bit rate for the GH fiber at 130 m, confirming that standard POFs are suitable for industrial and avionics data networks. In addition, even the high-aperture fibers are able to sustain 1 Gb/s for links of 30 m or less, and thus, to meet the typical requirements for domestic and automotive applications. Moreover, these bit rates could be raised by introducing modulation formats to increase spectral efficiency and forward error correction (FEC) techniques to permit BER values of up to 10^−3^, whereas the link reach could be pushed farther using receivers with higher responsivity at 520 nm.

Although previous results showed that the performance for the standard GH fiber was significantly better than for the heat-resistant BH [22], here we found only small differences between GH and BH, even with slight superiority of BH that we attribute to their different launching conditions, which was illustrated before by the differences between their 1-m EAFs. Another possible reason is that, in this work, we tested a recently acquired spool of BH fiber with improved characteristics. In addition, the duplex reel configuration can also affect the fiber properties, as it is necessary to split a segment at both ends, which can introduce additional disturbances in the power distribution of the GH fiber.

The lower graphs (see Figure 5c,d) show that there were small differences in the frequency domain performances obtained for red and green light with the GH fiber. From 50 m downwards, the fiber bandwidths obtained at 650 nm were always higher than those obtained at 520 nm, with the differences increasing for shorter fiber lengths. For 15 m, bandwidths of 729 and 1390 MHz were obtained for green and red light, respectively. Moreover, the maximum data rate was also higher with red light below 70 m, although the red link did not work above 85 m due to the low received power. From these results, we cannot discard that the causes of the differences between red and green were their diverse launching conditions and high variability, increased by the fiber duplex configuration. Therefore, we carried out the control experiment previously described to determine if there are significant differences between the frequency responses for 650 nm and 520 nm measured under the same conditions. The results are shown in the next subsection.

### 3.2. Frequency Responses for Red and Green Light

**Figure 6:**
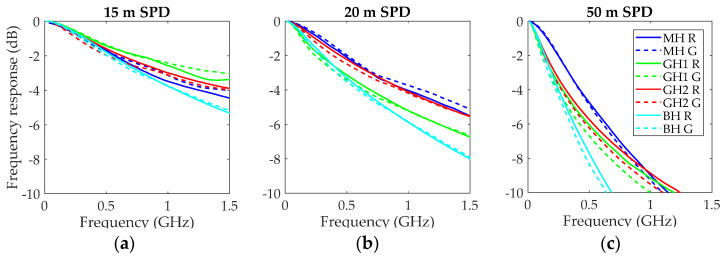
Normalized frequency responses obtained with a pigtail launch and the two laser diodes using the SPD receiver: (**a**) fiber length of 15 m; (**b**) fiber length of 20 m; (**c**) fiber length of 50 m.

We obtained the frequency responses with the experimental protocol described above, which was specifically designed to maintain similar launching conditions with both wavelengths by using a pigtail that was alternatively connected to the two laser diodes. In addition, the detection conditions were identical because the connection to the SPD receiver remained in place when changing sources. In these experiments, we tested the three fiber types for both sources using segments of three different lengths: 50, 20, and 15 m. The measurements were repeated twice, but in Figure 6, the results of only one session are shown. Normalized frequency responses for both wavelengths and the three fiber lengths are plotted on the left, middle and right graphs, respectively. In all graphs, a second set of measurements is shown for the GH fiber. These measurements were taken using the other fiber of the duplex segment in order to assess fiber variability.

The results show that, for all fiber types and lengths, there are only very small differences between the frequency responses with red and green light. However, with a few exceptions (15 and 20 m MH, and 15 m GH), the measurements with red light are above those obtained with green light. In the three exceptions, the curves show oscillations that can be attributed to small variations in the reference due to the changes at the connection of the RF cable from one mount to the other, which are necessary to perform both measurements. The S_21_ transmission parameter measurements were similar for short fibers and for the reference, so that any small alteration was magnified with the calculation of the fiber frequency response as its quotient (see Equation (1)). In addition, the differences between the two measurements for the GH fiber, particularly those obtained for the 20-m segment, are greater than for the measurements with red and green light, suggesting that fiber variability has more impact than the possible differences between wavelengths.

**Figure 7:**
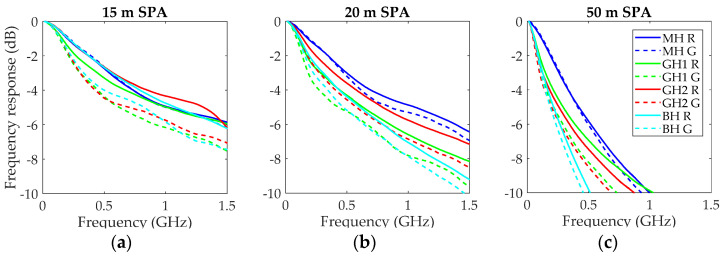
Normalized frequency responses obtained with a pigtail launch and the two laser diodes using the SPA receiver: (**a**) fiber length of 15 m; (**b**) fiber length of 20 m; (**c**) fiber length of 50 m.

At this point, it is important to note that the SPD receiver from Graviton was equipped with a lens to focalize the light onto its active area, so that it has a small aperture of 0.25 (14.5°). Thus, the SPD lens filtered out a high percentage of the fiber output power. The EAFs in Figure 2 show that this percentage was between 25% and 55% for GH and BH, and even less for MH. We suppose that this filtering effect could have been masking the differences between measurements with red and green light. For this reason, we also performed the control experiment using the SPA receiver, also from Graviton, whose lens has an aperture of 0.5 (30°), and thus, it is able to capture practically all the power that exits the fibers. Figure 7 shows the results using the SPA in the same way as in Figure 6.

A comparison of the corresponding graphs in Figure 6 and Figure 7 reveals that the frequency responses obtained with the SPD were flatter than those obtained with the SPA. This effect was not owed to the better response of the SPD, because its effect was taken into account by the reference in Equation (1). The reason was the spatial filtering due to the small aperture of the SPD receiver, which enhanced the frequency response, because it blocked power carried by middle and high order modes that, as they propagated through longer paths inside the fiber, introduced higher delays that degraded the fiber frequency response [23].

**Figure 8:**
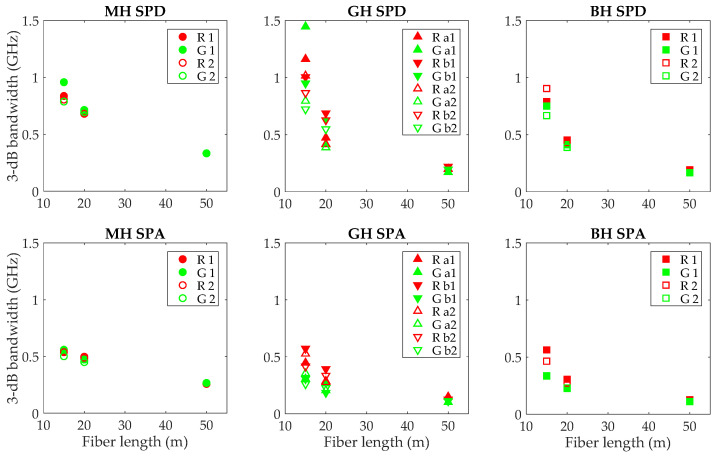
Evolution of the 3-dB bandwidth with length for the three fibers and the two receivers. Upper graphs: results obtained with the SPD; lower graphs: results obtained with the SPA; from left to right, data for the MH, GH and BH fibers, respectively.

Thus, the use of the SPA receiver with a higher aperture revealed larger differences between both wavelengths for the GH and BH fibers. On the other hand, for MH they are similar to those obtained with the SPD, because both receivers were able to capture most of its output power due to its lower NA. For GH and BH fibers, differences between wavelengths reduced with increasing length. In fact, at 50 m, they are similar to those obtained with the SPD, because the filtering was less effective as the power distributions were close to equilibrium with nearly complete mode mixing. In Figure 8, we represent the 3-dB bandwidths calculated from all the frequency responses obtained in the two sessions.

This figure confirms that the fiber bandwidths were better with red light for the two high-NA fibers, and that the differences decreased with fiber length, being practically negligible at 50 m. On the other hand, the figure reveals only insignificant differences in the bandwidths at red and green wavelengths for the MH fiber and all tested lengths. Therefore, we conclude that, at least for the high-NA fibers, modal dispersion is higher for green than for red light. We propose that one of the causes underlying the higher dispersion is the larger number of modes that the fiber is able to transmit at shorter wavelengths. For a SI fiber, the number of modes N is given by: ${N=V}^{2}/2$, with the normalized frequency, *V*, being defined as:

(2) $$V\;=\frac{2\pi }{\lambda }a\;\mathrm{NA}$$

where a is the fiber’s radius (approximately 500 µm for the PMMA POFs considered) and NA its numerical aperture. Thus, the number of modes is inversely proportional to the squared wavelength, implying an increase of 1.6 times when using the 520 nm laser diode. As the number of modes is also proportional to the square of the fiber NA, this explanation is consistent with the different behavior found for the low-NA MH fiber. In addition, we suggest that another contribution to the increase of modal dispersion can be the higher diffusion for shorter wavelengths. Diffusion in PMMA fibers has been attributed to Rayleigh and Mie scattering [15], whose dependence with wavelength is consistent with our findings. As Rayleigh scattering varies with λ^−4^, and Mie scattering approximately with λ^−2^, both effects are expected to be greater at 520 than at 650 nm [15,24]. Therefore, the combination of the increased number of modes and the stronger diffusion for the 520-nm wavelength explains the higher modal dispersion that was behind the behavior of our measured frequency responses.

## 4. Summary and Conclusions

We confirmed the lower attenuation of large-core PMMA step-index fibers of different numerical apertures at the 520-nm wavelength. In fact, an attenuation coefficient as low as 0.09 dB/m was found at this wavelength for the BH and GH fibers. On the other hand, our results suggest that green light introduces higher modal dispersion attributable to the higher number of modes combined with stronger diffusion that is consistent with the wavelength dependence of the predominant scatterings in PMMA. However, its impact on fiber bandwidth is not large, so transmission performance is not highly compromised. Therefore, the use of green optical sources is particularly interesting for long links where its lower attenuation is crucial, and the bandwidth is practically the same as measured with equivalent red sources. Actually, we found that all tested fibers are able to carry Fast-Ethernet data for 100 m, as required for industrial environments. Our results show that the differences in attenuation and frequency response between red and green light are smaller for MH than for the other fibers, consistent with its lower aperture. In fact, the maximum length achievable with this fiber is limited by attenuation, but on the other hand, its reduced dispersion allows reaching higher bit rates than any of the other fibers below 100 m. Thus, the MH fiber is suitable for high-capacity links demanded for domestic and automotive applications, as it supports Gigabit rates for up to 50 m. The heat-resistant BH fiber showed very good performance at 520 nm, reaching rates above 100 Mb/s at 100 m. In addition, its lower attenuation for green light can help to mitigate the problem of the stringent power budget in avionic networks. Moreover, the introduction of spectrally efficient modulation formats; the application of equalization and FEC techniques; and the use of receivers with higher responsivity for green light could increase these bandwidth-length products.

## Data Availability

The data presented in this study are available on request from the corresponding author.
